# Saúde na Copa: The World’s First Application of Participatory Surveillance for a Mass Gathering at FIFA World Cup 2014, Brazil

**DOI:** 10.2196/publichealth.7313

**Published:** 2017-05-04

**Authors:** Onicio Leal Neto, George Santiago Dimech, Marlo Libel, Wayner Vieira de Souza, Eduarda Cesse, Mark Smolinski, Wanderson Oliveira, Jones Albuquerque

**Affiliations:** ^1^ Epitrack Recife Brazil; ^2^ SingularityU Recife Chapter Recife Brazil; ^3^ Aggeu Magalhães Research Center Departament of Health Collective Recife Brazil; ^4^ Pernambuco Health Departament Recife Brazil; ^5^ Skoll Global Threats Fund Pandemics Team San Francisco, CA United States; ^6^ Aggeu Magalhães Research Center Departament of Public Health Recife Brazil; ^7^ Brazil's Ministry of Health General Coordination of Public Health Emergencies Response Brasilia Brazil; ^8^ Federal Rural University of Pernambuco Informatics Departament Recife Brazil

**Keywords:** mass gatherings, participatory surveillance, public health, epidemiology

## Abstract

**Background:**

The 2005 International Health Regulations (IHRs) established parameters for event assessments and notifications that may constitute public health emergencies of international concern. These requirements and parameters opened up space for the use of nonofficial mechanisms (such as websites, blogs, and social networks) and technological improvements of communication that can streamline the detection, monitoring, and response to health problems, and thus reduce damage caused by these problems. Specifically, the revised IHR created space for participatory surveillance to function, in addition to the traditional surveillance mechanisms of detection, monitoring, and response. Participatory surveillance is based on crowdsourcing methods that collect information from society and then return the collective knowledge gained from that information back to society. The spread of digital social networks and wiki-style knowledge platforms has created a very favorable environment for this model of production and social control of information.

**Objective:**

The aim of this study was to describe the use of a participatory surveillance app, Healthy Cup, for the early detection of acute disease outbreaks during the Fédération Internationale de Football Association (FIFA) World Cup 2014. Our focus was on three specific syndromes (respiratory, diarrheal, and rash) related to six diseases that were considered important in a mass gathering context (influenza, measles, rubella, cholera, acute diarrhea, and dengue fever).

**Methods:**

From May 12 to July 13, 2014, users from anywhere in the world were able to download the Healthy Cup app and record their health condition, reporting whether they were good, very good, ill, or very ill. For users that reported being ill or very ill, a screen with a list of 10 symptoms was displayed. Participatory surveillance allows for the real-time identification of aggregates of symptoms that indicate possible cases of infectious diseases.

**Results:**

From May 12 through July 13, 2014, there were 9434 downloads of the Healthy Cup app and 7155 (75.84%) registered users. Among the registered users, 4706 (4706/7155, 65.77%) were active users who posted a total of 47,879 times during the study period. The maximum number of users that signed up in one day occurred on May 30, 2014, the day that the app was officially launched by the Minister of Health during a press conference. During this event, the Minister of Health announced the special government program Health in the World Cup on national television media. On that date, 3633 logins were recorded, which accounted for more than half of all sign-ups across the entire duration of the study (50.78%, 3633/7155).

**Conclusions:**

Participatory surveillance through community engagement is an innovative way to conduct epidemiological surveillance. Compared to traditional epidemiological surveillance, advantages include lower costs of data acquisition, timeliness of information collected and shared, platform scalability, and capacity for integration between the population being served and public health services.

## Introduction

Mass gatherings (MGs) are situations that involve large populations that come together for specific causes related to leisure (eg, sports events, carnivals, and concerts), religion (eg, Hajj, World Youth Day), politics (eg, marches, protests, presidential inaugurations), or similar purposes [1-4]. By changing business, media, and public health environments, MGs create both new opportunities and novel risks [3-5].

During any MG, two changes in particular have the potential to significantly increase pressure on the local health care system. The first is the increase in simple contact between travelers (ie, tourists, athletes, workers, volunteers, press staff, authorities) and individuals in the local population [1-3]. This contact can promote both the introduction of diseases into the local population by visitors and the transmission of diseases to visitors through either visitor-to-visitor or native-to-visitor contact. The potential for transmission among visitors and between natives and visitors (both native-to-visitor and visitor-to-native) is further influenced by the increased mobility of people and goods made possible by the forces of globalization [3]. This mobility increases contact among individuals and goods from different epidemiological settings, promotes the exchange of potential threats to public health, and generates new risks [3,4,6,7]. Recent experiences with the persistence of dengue fever and increased circulation of Zika virus [8-10] and Chikungunya fever have made the threat of infectious disease transmission a tangible reality that requires rapid detection and response preparation (ie, including case findings, vaccine availability, diagnostic procedures, medical services, epidemiological research, medicines, and guidance for the public and professionals) [3,4,7]. A second MG-associated change that has the potential to significantly increase pressure on the local health care system is the size of the MG itself, which can impact routine demand not only for health, but for all public services, especially security and transportation [3,4,6].

The revised International Health Regulations (IHRs) 2005 required states to develop, strengthen, and maintain the capabilities to detect, assess, notify, and report risk events to international public health authorities, including situations resulting from MGs [11]. Additionally, the 2005 IHRs established parameters for event assessments and notifications that may constitute public health emergencies of international concern [12-14]. These requirements and parameters opened up space for the use of nonofficial mechanisms (eg, websites, blogs, and social networks) and technological improvements of communication that can streamline the detection, monitoring, and response to health problems, and thus reduce damage caused by these problems [15-19]. Specifically, the revised IHRs created space for participatory surveillance to function, in addition to the traditional surveillance mechanisms of detection, monitoring, and response [20-27]. Participatory surveillance is based on crowdsourcing methods that collect information from society and then return the collective knowledge gained from that information back to society. The spread of digital social networks and wiki-style knowledge platforms has created a very favorable environment for this model of production and social control of information [18,19,28-31].

The aim of this study was to describe the use of a participatory surveillance app, Healthy Cup, for the early detection of acute disease outbreaks during the Fédération Internationale de Football Association (FIFA) World Cup 2014. The main health outcomes (ie, outcomes related to infectious diseases) that have been associated with MG sporting events like this are respiratory, cardiovascular, and gastrointestinal (ie, diarrhea) [32-36]. Respiratory outcomes are mainly associated with viruses that can spread easily between individuals, cardiovascular outcomes are usually associated with the emotional stress that fans experience during sports events, and gastrointestinal outcomes are often associated with the expansion of the informal food trade that typically occurs during MG events (eg, foods being sold on the street) and the desire of tourists to sample local cuisine, which may not be well tolerated by travelers’ bodies. Our focus was on these three main infectious disease outcomes, specifically respiratory, diarrheal, and rash syndromes related to diseases that are considered important in an MG context (ie, influenza, measles, rubella, cholera, acute diarrhea, and dengue fever).

## Methods

From May 12 to July 13, 2014, users from anywhere in the world were able to download the Healthy Cup app and record their health condition, reporting whether they were *good*, *very good*, *ill*, or *very ill*. For users that reported being *ill* or *very ill*, a screen with a list of 10 symptoms was displayed. Participatory surveillance allows for the real-time identification of aggregates of symptoms that indicate possible cases of infectious diseases. Table 1 lists the symptoms, and syndromes and diseases associated with these symptoms, that were included in the Healthy Cup app. In addition to these 10 symptoms, the app also had 2 additional queries regarding contact chain (eg, “I got in touch or know someone with some of these symptoms in the last 7 days”) and severity of the symptoms (“I looked for a health care service”).

**Table 1:** Symptoms, syndromes, and diseases searched using Healthy Cup app.

Symptoms	Syndromes			Diseases					
	Respiratory	Diarrheal	Rash	Influenza	Measles	Rubella	Cholera	Acute diarrhea	Dengue
Fever	X	X	X	X	X	X	-	X	X
Cough	X	-	X	X	X	X	-	-	-
Sore throat	X	-	-	X	-	-	-	-	-
Nausea	-	X	-	-	-	-	-	X	-
Joint pain	-	-	X	-	-	-	-	-	X
Headache	-	-	X	-	-	-	-	-	X
Diarrhea	-	X	-	-	-	-	X	X	-
Rash	-	-	X	-	X	X	-	-	X
Bleeding	-	-	X	-	-	-	-	-	X
Shortness of breath	X	-	-	X	-	-	-	-	-

Healthy Cup was developed by a partnership between the Secretariat of Health Surveillance (Brazil’s Ministry of Health), Skoll Global Threats Fund, and Epitrack eHealth. The app was designed on an open source platform for use on mobile devices. The platform was developed as a hybrid app for both iOS and Android operating systems that could be accessed by anyone using an iOS or Android smartphone, or as a Web app in any Internet browser. The iOS native and Web apps were developed using PhoneGap (built with JavaScript, HTML5 and CSS) [37]; the Android app was developed in native language. The mobile and Web apps use external interface capabilities to support Application Program Interface (API) Google Places (for location of nearby hospitals and pharmacies), Google Maps API (for user navigation to points of interest and viewpoints on the dashboard), and Twitter API (for streaming of the Ministry of Health profile) [38,39]. We used a MySQL-type server developed in PHP language, and we managed the database using phpMyAdmin.

The Healthy Cup project was hosted throughout the study period by Dreamhost [40], and its code and versioning used GitHub. To ensure safety of the platform, we also set up a virtual private server with 60 gigabytes of storage, 2 gigabytes of random-access memory, and unlimited bandwidth. Both reports and registers had geolocation features, in which the system captured the coordinates related to each use. This function was implemented following these standards to acquire geolocation data: (1) asking the permission of the users; (2) starting up a cron job to get the coordinates; and (3) inserting this coordinate for each respective event, whether it was a report or register.

## Results

From May 12 through July 13, 2014, there were 9434 downloads of the Healthy Cup app and 7155 (75.84%) registered users. Among the registered users, 4706 (4706/7155, 65.77%) were active users who posted a total of 47,879 times during the study period. Of these posts, 89.43% (42,818/47,879) reported no symptoms. One symptom was reported in 3173 posts (3173/47,879, 6.63%); one or more symptoms were reported in 5329 posts (5329/47,879, 11.13%; with an average of 1.8 per post); five or more symptoms were reported in 220 posts (220/47,879, 0.46%); and all 10 symptoms were reported in 99 posts (99/47,879, 0.21%; see Table 1 and Table 2).

**Table 2:** Distribution of the posts, symptoms profile, and syndromes by host city. Saúde na Copa 2014 percentages for posts are in relation to total posts among all host cities; percentages for symptoms are in relation to host city posts.

Host city	Posts, n (%)	With symptoms, n (%)	Diarrhea syndrome, n (%)	Respiratory syndrome, n (%)	Rash Syndrome, n (%)
Belo Horizonte	1133 (4.32)	128 (11.30)	7 (5.5)	12 (9.4)	5 (3.9)
Brasilia	7951 (30.33)	573 (7.20)	14 (2.4)	37 (6.5)	7 (1.2)
Cuiabá	1109 (4.23)	173 (15.60)	1 (0.6)	15 (8.7)	0 (0.0)
Curitiba	824 (3.14)	92 (11.2)	6 (6.5)	9 (9.8)	2 (2.2)
Fortaleza	1519 (5.80)	174 (11.45)	6 (3.4)	9 (5.2)	3 (1.7)
Manaus	985 (3.76)	136 (13.8)	4 (2.9)	12 (8.8)	2 (1.5)
Natal	938 (3.58)	77 (8.2)	2 (2.6)	4 (5.2)	0 (0.0)
Porto Alegre	925 (3.53)	113 (12.2)	6 (5.3)	7 (6.2)	3 (2.7)
Recife	4316 (16.47)	282 (6.53)	5 (1.8)	9 (3.2)	1 (0.4)
Rio de Janeiro	3069 (11.70)	348 (11.34)	15 (4.3)	28 (8.0)	13 (3.7)
Salvador	1324 (5.04)	230 (17.38)	5 (2.2)	19 (8.3)	2 (0.9)
São Paulo	2125 (8.10)	358 (16.84)	16 (4.5)	37 (10.3)	5 (1.4)
Total	26,218 (100.00)	2684 (10.24)	87 (3.2)	198 (7.4)	43 (1.6)

**Figure 1:**
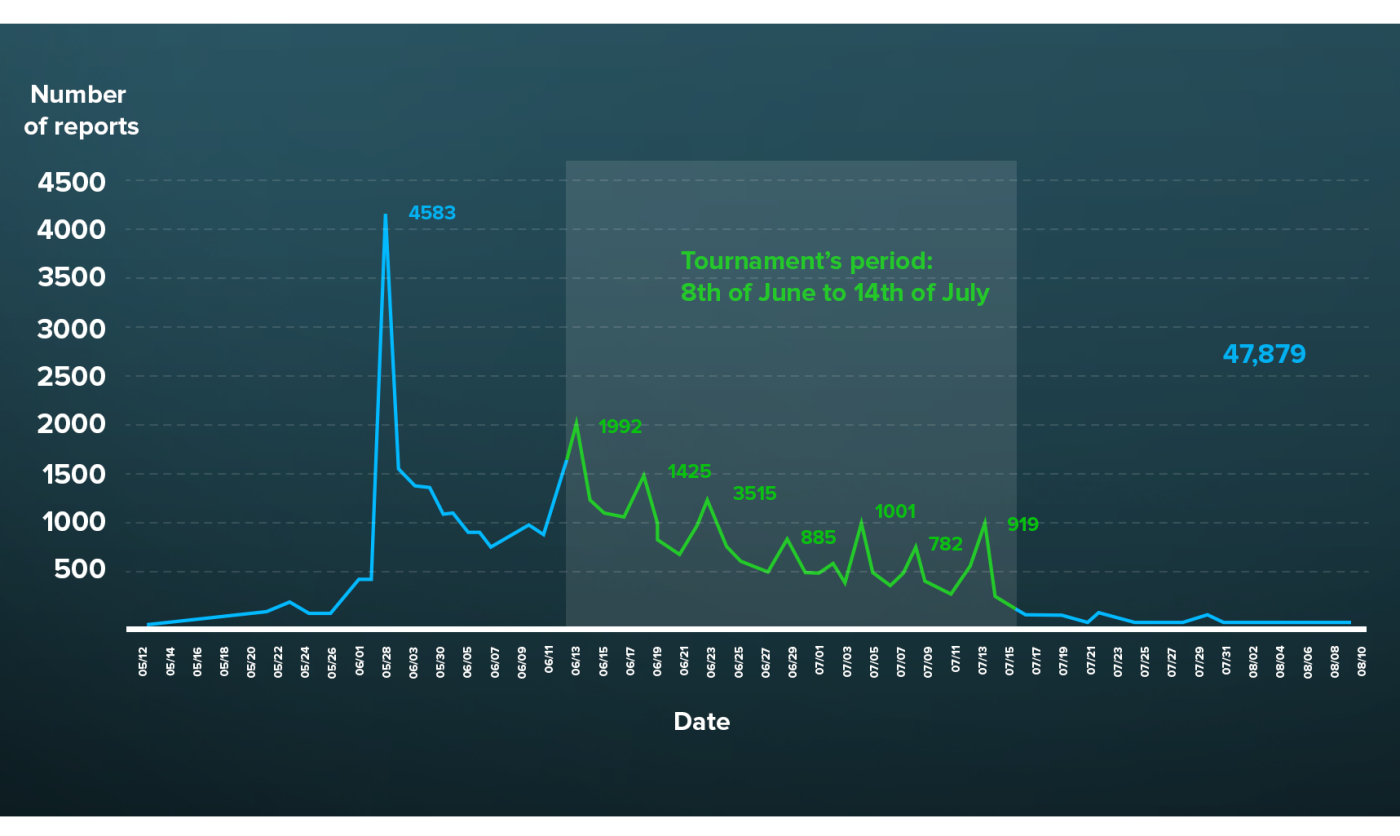
Temporal distribution of posts by dates, Saúde na Copa, 2014.

The maximum number of users that signed up in one day occurred on May 30, 2014, the day that the app was officially launched by the Minister of Health during a press conference. During this event, the Minister of Health announced the special government program *Health in the World Cup* on national television media (Figure 1). On that date, 3633 logins were recorded, which accounted for more than half of all sign-ups across the entire duration of the study (50.78%, 3633/7155). Based on cost reduction efforts by the Brazilian government, this was the only advertising action that was undertaken for the app.

**Figure 2:**
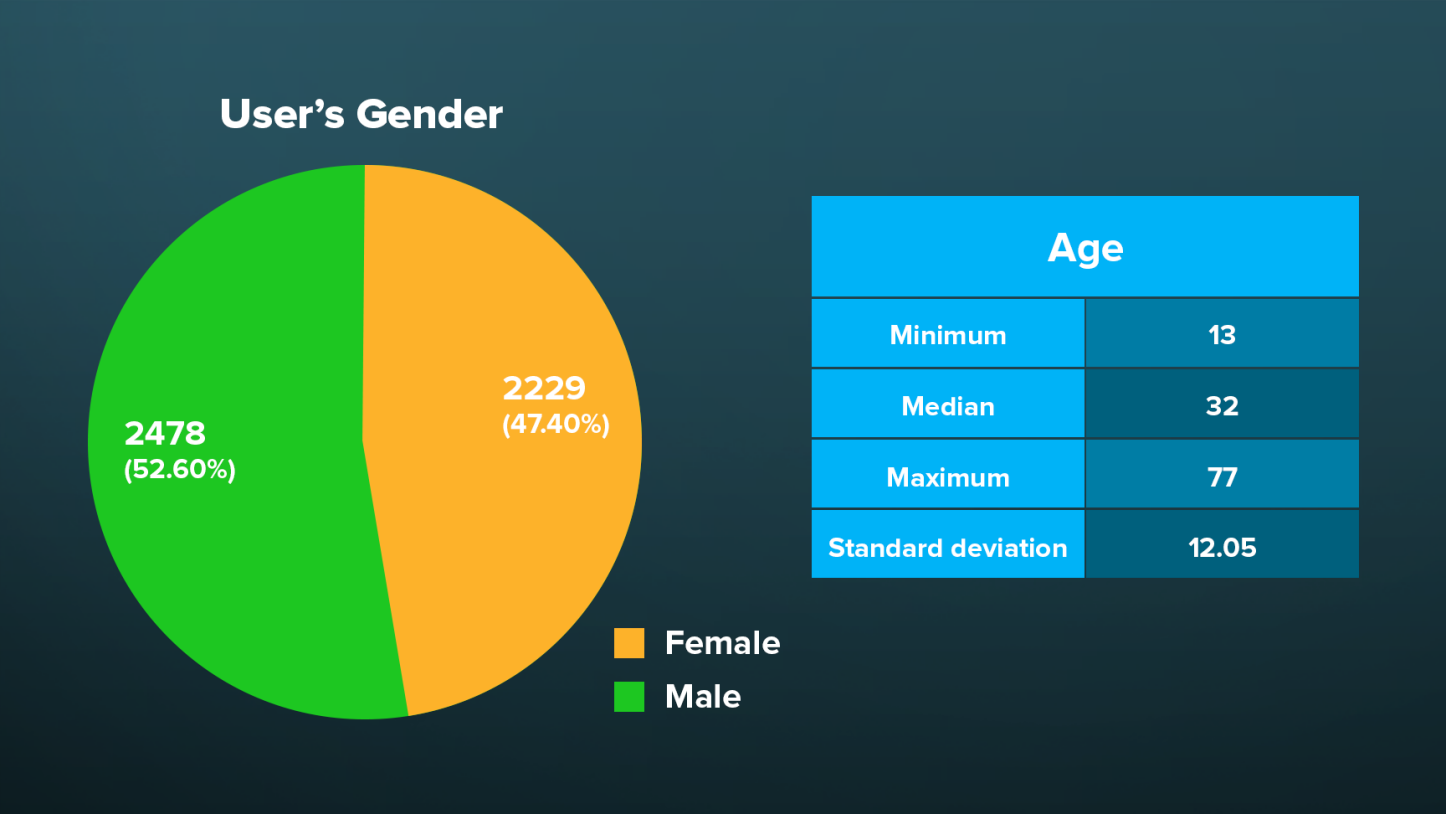
Distribution of users according to sex and age, Saúde na Copa, 2014.

Most of the active users (3526/4706, 74.95%) installed the app on Android mobile devices, 1167 (1167/4706, 24.80%) were on Apple iOS mobile devices, and 13 (13/4706, 0.28%) were on desktop computers. Slightly more than half of all users were male (2478/4706, 52.66%). Users ranged in age from 13 to 77 years with a median of 32 years (only individuals aged 13 years and older were allowed to use the app; see Figure 2). Due to privacy rules, we were not allowed to collect nationality data. A total of 4661 users (4661/4706, 99.04%) preferred the app in Portuguese, 34 (34/4706, 0.72%) in Spanish, and 12 (12/4706, 0.25%) in English.

**Figure 3:**
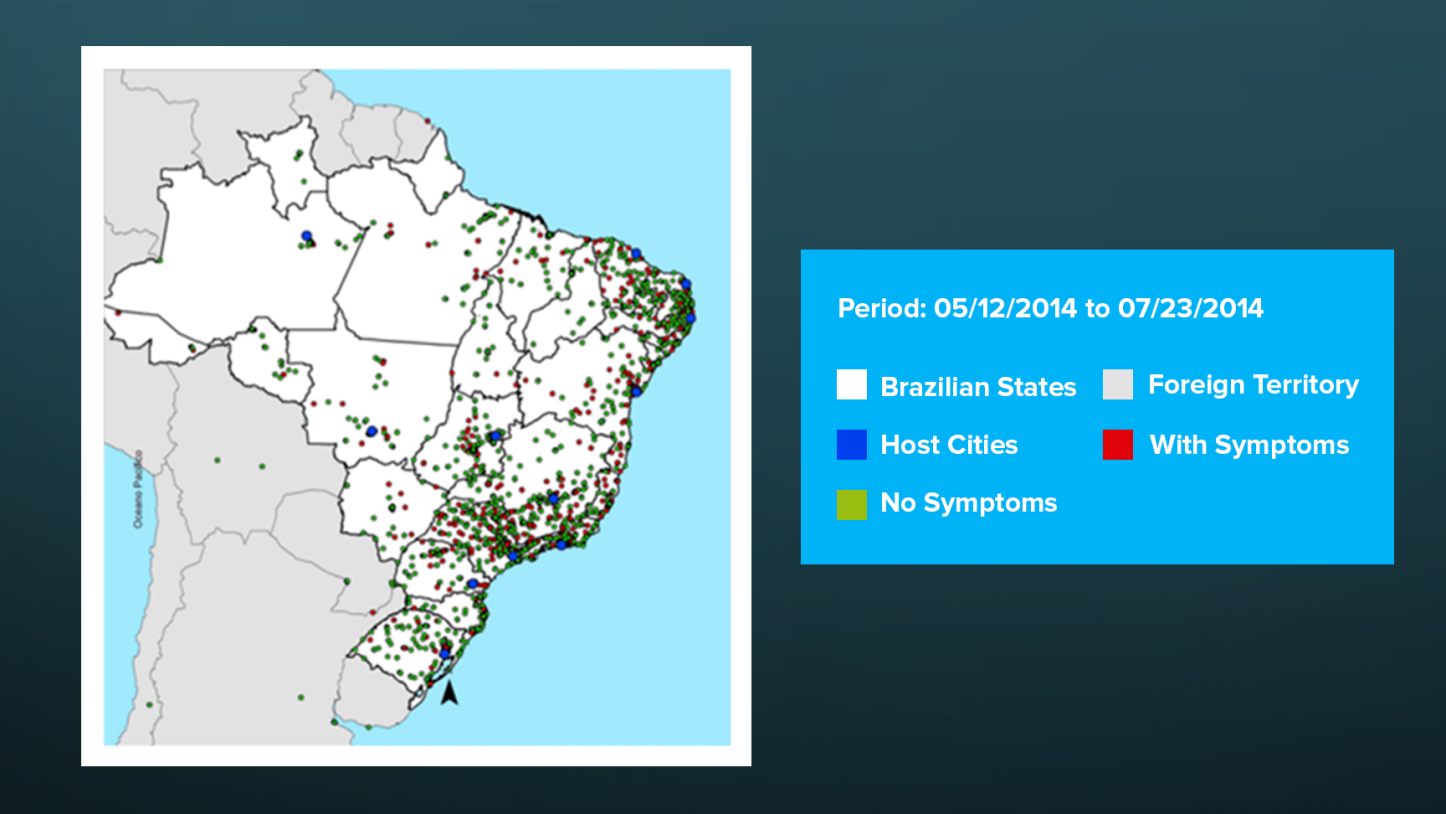
Spatial distribution of posts with and without symptoms during the period of launch of Saúde na Copa app until the end of the championship.

The intensity of participation in relation to the match dates of the Brazilian team and the World Cup calendar are illustrated in Figure 3 and Table 3, respectively. It should be emphasized that on the days the Brazilian team played (Figure 1) and in the second phase of the World Cup matches (Table 3), push notifications were sent to users through the app.

**Table 3:** Distribution of reports according to World Cup Saúde na Copa, 2014.

World Cup timeframe	All posts (reports)	% (of total posts)	Posts with symptoms	% (of all posts during timeframe)
1. Pre-World Cup (May 12 - June 11)	19,737	41.22	3490	17.68
2. Group phase (June 12 - 27)	16,868	35.23	1241	7.36
3. Eighth finals (June 28 - July 3)	3762	7.86	217	5.77
4. Fourth finals (Jul 4 - 7)	2438	5.09	103	4.22
5. Semifinals (Jul 9 - 11)	1910	3.99	128	6.70
6. Finals (Jul 12 and 13)	1533	3.20	98	6.39
7. Post-World Cup (Jul 14 - 23)	1631	3.41	82	5.03
Total	47,879	100.00	5359	11.19

Upon user authorization and if the device had an active global positioning system, the app automatically recorded the location of each post. Only 6.00% (2,824/47,879) of posts provided no location information. More than half of all posts were recorded in World Cup host cities (26,218/47,879, 54.76%; Table 2), 37.90% (18,146/47,879) were elsewhere in Brazil, and 1.40% (670/47,879) were in other countries. Table 2 also displays the locations with the highest number of known posts: Brasília (30.33%, 7951/26,218), Recife (16.46%, 4316/26,218), and Rio de Janeiro (11.71%, 3069/26,218), which together accounted for more than half (54.76%, 26,218/47,879) of all posts located in World Cup host cities. Among the 26,218 posts in World Cup host cities, the greatest number of symptoms were recorded in Brasilia (573 posts with symptoms), São Paulo (358 posts with symptoms), and Rio de Janeiro (348 posts with symptoms). Also among the 26,218 host city posts, the greatest frequency of symptoms proportionally were recorded in Salvador (17.38%, 230/1324), São Paulo (16.84%, 358/2125), and Cuiabá (15.60%, 173/1109).

Of the three syndromes detected by Healthy Cup (ie, respiratory, diarrheal, and rash), respiratory syndrome occurred with the greatest frequency based on reported symptoms. The greatest number of posts of respiratory syndromes were reported in São Paulo (n=37), Brasilia (n=37), and Rio de Janeiro (n=28). The highest frequencies of rash syndrome (ie, percentage of posts signaling rashes) were reported in Belo Horizonte (3.9%, 5/128), Rio de Janeiro (3.7%, 13/348), and Porto Alegre (2.7%. 3/113). The greatest number of diarrheal syndromes were reported in São Paulo (n=16), Rio de Janeiro (n=15), and Brasilia (n=14). The highest frequencies of diarrheal syndrome were reported in Curitiba (6.5%, 6/92), Belo Horizonte (5.5%, 7/128), Minas Gerais (5.5%, 7/128), and Porto Alegre (5.3%, 6/113). Finally, the greatest number of rash syndromes were reported in Rio de Janeiro (n=13), Brasilia (n=7), São Paulo (n=5), and Belo Horizonte (n=5).

## Discussion

Although the initial impact of the media was remarkable, with the greatest number of users signing up for Healthy Cup on the day the app was officially launched and first advertised on national television (Figure 1), this impact was not sustained even after 7 days of intense media (eg, television and websites) news about the app. The fact that the median age among those who signed up was 30 years suggests that this age group was more exposed to Healthy Cup promotion through television and website news [21,24,41,42]. The limited participation of foreigners (less than 1%) may reflect the limited language options of the app (English and Spanish) [24].

The increased number of posts during the Brazilian matches may be related to the sending of push notifications before those matches, which reminded participants to use the app [24,29]. The host city with the highest concentration of posts was Brasilia (30.33%, 7951/26,218), which may be related to the fact that Brasilia had the highest number of local news reports about the app.

More than half of all posts were recorded in World Cup host cities (54.76%, 26,218/47,879), suggesting that the app can be used as a tool to identify potential alerts for outbreaks associated with MGs [31-36]. The remaining 45.24% of posts (21,661/47,879) were recorded in cities elsewhere in Brazil (ie, other than the host cities of the World Cup), illustrating rapid penetration of the app across the country, even in states not hosting the FIFA World Cup 2014 [24].

Posts with six or more reported symptoms that were incompatible with the sought-after syndromes were considered *spam reports*. The higher prevalence of posts reported during the first two weeks (Figure 1) may be related to curiosity about this new type of tool being used in public health, with users wanting to record their health situation even if they were not showing symptoms [24].

Respiratory syndromes were reported more often than any other (Table 2), suggesting that the tool may have the capacity for early detection of epidemiological changes associated with influenza [41]. It is noteworthy that reports of bleeding and rash showed a high frequency of demand for health services [43,44].

The criteria used to classify syndromes (ie, based on parameters used by the Ministry of Health’s Secretariat of Health Surveillance) may have underestimated the number of users with any of the three syndromes (ie, respiratory, diarrheal, or rash) [45]. However, during this same time period, the official Health Surveillance System (at the Ministry of Health) routinely used by the Integrated Health Center of Joint Operations did not identify the occurrence of any public health emergency events (ie, syndromic clusters) warranting intervention. Thus, data and findings from the Healthy Cup platform were validated by traditional sources.

### Conclusions

Participatory surveillance through community engagement is an innovative way to conduct epidemiological surveillance. Compared to traditional epidemiological surveillance, the advantages of participatory surveillance include its lower cost for data acquisition, timeliness of information collected and shared, platform scalability, and capacity for integration between the population being served and public health services.

The pilot of the Healthy Cup app during the FIFA World Cup 2014 allowed us to evaluate the potential for participatory surveillance in Brazil. Based on our results, participatory surveillance appears to have the potential to become a routine component of national health surveillance and to help improve the early detection of outbreaks and epidemics, timely intervention, and risk minimization. The Healthy Cup platform in particular appears to be sensitive to multiple symptoms and syndromes associated with a range of potential threats.

We also learned several lessons from our piloting experience, including the idea that investment in communication, marketing, and advertising is necessary to penetrate multiple social strata (eg, different age groups) and to reach as many users as possible. Relying on spontaneous media (ie, news and nonpaid ads) and press conferences alone limited use of the app to consumer groups that seek out this specific type of information. Investment in digital media could create a great opportunity to not only boost the number of users, but also enhance the engagement of users.

Another lesson learned is the need for reciprocity. Citizens may feel more motivated to participate if they receive something in return, such as information about diseases that are being reported in their area. This information could be sent back to users on maps or via specific screens within the app. Providing population-level information in return for individual participation could ensure continuous engagement with the app, which would improve data quality. The value of reciprocity should be tested in future MG participatory surveillance scenarios.

The Healthy Cup app served as the basis for the Guardians of Health (*Guardiões da Saúde*) participatory surveillance app that was used during the Olympic and Paralympic games in Brazil. An additional lesson learned through use of Guardians of Health is the importance of the need for clear expectations of a government’s role in participatory surveillance. Some governments may not have dedicated teams to examine and interpret data generated through participatory surveillance. This issue underscores the importance of designing an intuitive platform that generates easily-visualized data.

## Abbreviations

API: Application Program Interface
FIFA: Fédération Internationale de Football Association
IHR: International Health Regulation
MG: mass gathering
